# Magnesium Regulation Increases the Content of Characteristic Volatile Compounds and Enhances the Intensity of Odor Characteristics in Tea Tree Leaves

**DOI:** 10.3390/foods14061043

**Published:** 2025-03-19

**Authors:** Jianghua Ye, Qiqi Weng, Yulin Wang, Weiting Cheng, Junbin Gu, Qi Zhang, Bitong Zhu, Qiyan Liu, Xiaoli Jia, Juanying Wang, Haibin Wang

**Affiliations:** 1 College of Tea and Food, Wuyi University, Wuyishan 354300, China; jhye1998@wuyiu.edu.cn (J.Y.);; 2 College of Life Science, Longyan University, Longyan 364012, China; 3 College of Horticulture, Fujian Agriculture and Forestry University, Fuzhou 350002, China; 4 Institute of Agro-Bioengineering, Guizhou University, Guiyang 550025, China

**Keywords:** tea tree, magnesium regulation, characteristic volatile compounds, odor characteristics

## Abstract

Magnesium plays an important role in regulating the growth and quality of tea trees. However, the effect of magnesium regulation on changes in the aroma quality of tea tree leaves remains unknown. In this study, the volatile compounds of tea leaves under different magnesium concentrations were determined to obtain the characteristic volatile compounds that changed significantly and their odor characteristics and to explore the effect of magnesium regulation on the aroma quality of tea leaves. The results showed that magnesium significantly enhanced the content of 15 characteristic volatile compounds in tea tree leaves, especially heptyl formate and t-geraniol. The 15 characteristic volatile compounds mainly showed five kinds of odor characteristics, such as fruity, green, floral, pungent, woody, and burnt; magnesium regulation critically increased floral, fruity, and green odor characteristics. This study lays an important foundation for the application of exogenous magnesium ions to regulate tea aroma quality.

## 1. Introduction

In China, tea trees play a pivotal role as a key economic plant to promote the development of Chinese agricultural economy [1]. The growth of tea trees depends on the uptake of several elements to ensure their normal physiology and development [2]. Among them, magnesium plays a crucial role as one of the essential elements for tea tree growth [3]. According to a previous study, maintaining magnesium ion content in plants in the range of 1.5~3.5 mg/g is most favorable for plant growth [4]. And proper application of magnesium fertilizer has been shown to be effective in enhancing plant yield [5]. It has been reported that increased application of magnesium improves the efficiency of nutrient uptake and utilization by the root system of tea trees, which in turn increases the annual yield of tea by more than 15.8% or more [6]. The economic benefits of tea are closely related to its yield and quality, so improving tea quality while ensuring yield is essential to improving its economic benefits [7]. Magnesium plays an important role in the synthesis and distribution of secondary metabolites in tea tree leaves. However, while increasing magnesium supply enhances tea yield, it may lead to decreases in catechins, total phenols, and total free amino acids in tea leaves [8]. Chen et al. [9] found that adequate magnesium supply favored the growth of the tea tree root system and increased the leaf starch content but inhibited the accumulation of secondary metabolites, thus affecting the quality of tea tree leaves. Similarly, Xu et al. [10] found that magnesium fertilization could promote the accumulation of theanine and reduce the content of tea polyphenols in tea tree leaves, which made the tea taste more refreshing and reduced bitterness and astringency. Zhang et al. [11] explored the effect of magnesium on tea quality with the help of transcriptomics technology and concluded that increasing the magnesium supply would inhibit the accumulation of theanine and caffeine, which in turn was detrimental to the overall quality of tea. Taken together, several studies have shown that although magnesium helps to increase tea yield, it adversely affects the synthesis of major biochemical quality components of tea, such as tea polyphenols, catechins, and caffeine, which may indirectly hinder the enhancement of tea quality.

Aroma, as an important characteristic of tea quality, plays a crucial role in the formation and grading of tea quality [12]. In research on the effect of magnesium on tea quality, many scholars have focused on changes in tea biochemical quality indexes after magnesium regulation, which is the basis for evaluating tea quality [8,9,11]. However, little research has been reported on the effect of magnesium regulation on tea aroma quality. The formation of tea aroma characteristics and its intensity are closely related to the type and content of volatile compounds in tea tree leaves [13]. By determining the content of volatile compounds in tea tree leaves, the aroma quality of tea can be effectively evaluated [14]. Gas chromatography-mass spectrometry (GC-MS) has shown significant advantages in the determination of volatile compounds due to its excellent performance, and it has been widely used in the analysis of volatile compounds in tea [15]. For example, Fu et al. [16] used GC-MS to analyze the volatile compounds of 22 types of fresh leaves of oolong tea and revealed the main substances responsible for the formation of the aroma differences between different varieties of oolong tea. Zhang et al. [17] analyzed the volatile compounds of different aroma types of Longjing tea by GC-MS and identified the key volatile compounds that differentiated between the different aromas. Accordingly, this study used HS-SPME and GC-MS to analyze the type and content of volatile compounds in tea tree leaves after magnesium regulation at different concentrations and further identified the characteristic volatile compounds and their odor characteristics that changed significantly after magnesium regulation, aiming to explore the effects of magnesium regulation on the aroma quality of tea and provide a solid theoretical foundation for the enhancement of the aroma quality of tea through the regulation of exogenous magnesium ions.

## 2. Materials and Methods

### 2.1. Chemical Reagents and Instruments

The chemical reagents used in this study, such as KNO_3_ (Sinopharm Chemical Reagent Co., Ltd., Beijing, China), (NH_4_)_2_SO_4_ (Sinopharm Chemical Reagent Co., Ltd., Beijing, China), K_2_SO_4_ (Sinopharm Chemical Reagent Co., Ltd., Beijing, China), FeSO_4_ (Sinopharm Chemical Reagent Co., Ltd., Beijing, China), Al_2_(SO_4_)_3_ (Sinopharm Chemical Reagent Co., Ltd., Beijing, China), KH_2_PO_4_ (Sinopharm Chemical Reagent Co., Ltd., Beijing, China), CaCl_2_ (Sinopharm Chemical Reagent Co., Ltd., Beijing, China), and MgSO_4_ (Sinopharm Chemical Reagent Co., Ltd., Beijing, China) were analytically pure. The instruments used were a fully automated solid-phase microextractor (HS-SPME, SPME Arrow, CTC Analytics AG, Zwingen, Switzerland), headspace vials (Agilent, Palo Alto, CA, USA), DVB/CWR/PDMS extraction heads (Agilent, Palo Alto, CA, USA), an analyzing tube aging device (Fiber Conditioning Station, CTC Analytics AG, Zwingen, Switzerland), gas chromatography (GC, 8890B, Agilent, CA, USA), mass spectrometry (MS, 7000D, Agilent, CA, USA), and DB-5MS capillary column (30 m × 0.25 mm × 0.25 μm, Agilent, CA, USA).

### 2.2. Experimental Design and Sample Collection

In this study, one-year-old tea tree seedlings (Rougui, about 30 cm in height and 0.3 cm in diameter) with uniform growth were selected. Seedlings were transplanted to complete nutrient solution for 45 d and then used in the magnesium regulation experiment. The complete nutrient solution was slightly modified from the formulation of Sun et al. [18], and the concentrations of KNO_3_, (NH_4_)_2_SO_4_, K_2_SO_4_, FeSO_4_, Al_2_(SO_4_)_3_, KH_2_PO_4_, CaCl_2_, and MgSO_4_ were 125, 187.5, 25, 16, 200, 100, 100, and 100 μmol/L, respectively. The pH of the nutrient solution was adjusted to 4.5.

The root systems of the recovered tea tree seedlings were rinsed with deionized water and then transplanted to three different magnesium concentrations in complete nutrient solution for culture. Three independent replicates were established for each magnesium concentration, and magnesium concentrations in the three different treatments were 0 mmol/L (ML), 0.4 mmol/L (MM), and 0.8 mmol/L (MH), while the concentrations of other ions were consistent with the above complete nutrient solution. After transplanting, the tea trees were placed in an artificial climatic chamber for cultivation, with continuous aeration of the culture solution for 24 h per day, a light period of 12 h (8:00 a.m.~20: 00 p.m.), an intensity of 1500 lux, a temperature of 25 °C, and a humidity of 75%. After magnesium regulation, tea tree seedlings were treated for 21 d. During this period, the same culture solution was changed every 7 days. At the end of magnesium regulation, one bud and two leaves of tea tree seedlings were collected and immediately stored in liquid nitrogen for extraction and determination of volatile compounds, with three independent replicates for each treatment.

### 2.3. HS-SPME Extraction of Volatile Compounds

Samples of tea trees treated with different magnesium concentrations were ground and mixed using liquid nitrogen, respectively. For each sample, 500 mg was weighed and placed in a headspace bottle, and 10 mL of saturated NaCl solution was added to collect tea volatile compounds using fully automated HS-SPME. Three independent replicates were performed for each sample. The extraction method was described as follows: the extraction head was a 120 µm DVB/CWR/PDMS solid-phase microextraction head, the extraction head was placed in a resolving tube aging device for 2 h before sampling, and the temperature was set at 250 °C. The volatile compounds were collected by placing the headspace vials containing the tea tree leaf samples in a thermostatic water bath at 60 °C, oscillating for 5 min. The extraction head was immediately inserted into the headspace bottle, and the extraction time was 15 min, and the sample was then used for gas chromatography-mass spectrometry (GC-MS) determination and analysis [19].

### 2.4. Determination of Volatile Compounds by GC-MS

An Agilent 8890B gas chromatograph (GC) coupled with a 7000D mass spectrometer (MS) was used to determine volatile compounds in tea leaves. The capillary column used for GC was an Agilent DB-5MS and the carrier gas was helium. First, the solid-phase microextraction head was placed in the inlet port of the GC and desorbed for 5 min, and the temperature was set at 250 °C. The flow rate of the carrier gas for the GC was 1.2 mL/min, and the sample was injected without splitting, with a solvent delay of 3.5 min. The programmed heating mode of the GC was set to first hold the sample at 40 °C for 3.5 min, and then linearly warm it up to 100 °C at 10 °C/min, and then linearly warm it up to 180 °C at 7 °C/min, and then linearly warm it up to 280 °C at 25 °C/min for 5 min. MS was performed by electron bombardment of the ion source, in which the temperature of the ion source was set at 230 °C, the temperature of the mass spectrometry connector was set at 280 °C, the temperature of the quadrupole was set at 150 °C, and the electron energy was 70 eV. The mass range of the mass spectrometry scan was in the range of 10–1000 *m*/*z* and the scan time was 30 min. MS was performed in selective ion detection mode, with qualitative and quantitative ions scanned precisely.

After GC-MS determination, the sample data were collected and processed using MassHunter software (B 08.00). This was done by selecting 2~3 qualitative ions for each compound and comparing them with the NIST20 mass spectrometry database. A compound was characterized if all qualifying ions appeared in the mass spectra of the compound after subtracting the background and the retention time was consistent with the database standard [20]. After characterization of the compound, a quantitative ion was selected, integrated, and corrected, and the compound was quantified based on the obtained peak area [21].

### 2.5. Statistical Analysis

Raw data on volatile compounds of tea leaves were initially organized and counted using Excel 2020 and subsequently unified for in-depth analysis and graphing with the help of Rstudio software (v 4.2.3) [22]. Box plots for volatile compound content analysis were plotted using gghalves 0.1.4; multi-group volcano plots between ML, MM, and MH were generated through the combined application of ggplot2 and ggnewscale; K-means clustering analysis of differential volatile compounds was executed using stats 4.2.0; OPLS-DA models were constructed for ML, MM, and MH with the help of ropls and mixOmics and the corresponding graphs were produced; bubble feature plots of volatile compounds were produced using ggplot2 3.4.0; Mulberry diagram analysis for volatile compound classification was implemented using networkD3 0.4.1; TOPSIS analysis of volatile the compound contribution rates was performed by dplyr 1.1.4; odor wheels for characteristic volatile compounds were jointly produced using circlize version 0.4.15 and vegan version 2.6.4; and conventional radar plots and principal component analysis plots were produced relying on fmsb 0.7.6 and ggbiplot 0.55, respectively. To assess variability between samples or indicators, variance analysis and paired student’s t-tests were used, and differences were considered to reach a significant level when *p* < 0.05.

## 3. Results and Discussion

### 3.1. Effect of Magnesium Regulation on Volatile Compounds of Tea Tree Leaves

**Figure 1:**
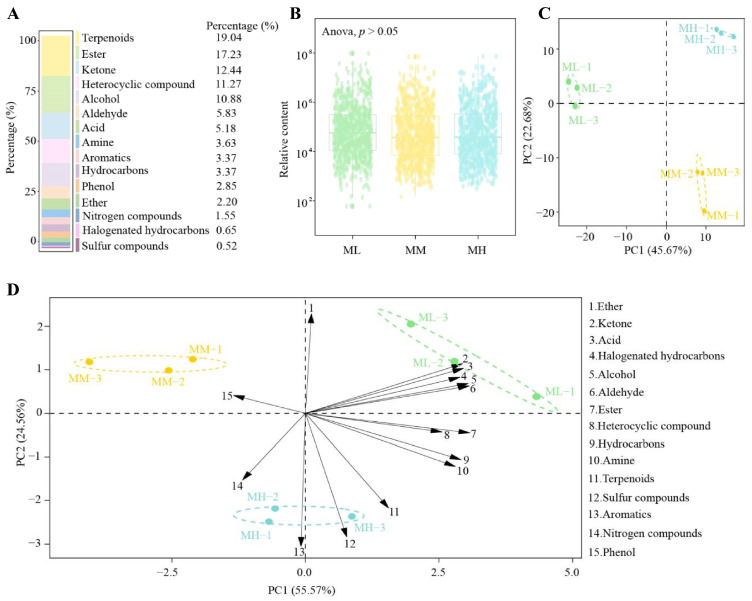
Effect of magnesium regulation on the type and content of volatile compounds in tea tree leaves. Note: ML: Mg^2+^ concentration 0 mmol/L; MM: Mg^2+^ concentration 0.4 mmol/L; MH: Mg^2+^ concentration 0.8 mmol/L; (**A**) Types of volatile compounds of tea tree leaves and their percentage of quantity; (**B**) Effect of magnesium regulation on the volatile metabolite content of tea tree leaves; (**C**) Principal component analysis of different samples under magnesium regulation; (**D**) Principal component analysis of volatile compounds of different samples after categorization under magnesium regulation.

Volatile compounds, which are mainly derived from secondary metabolites of tea trees, are the main compounds that form the aroma characteristics of tea [23]. Elemental supply plays an important role in the synthesis of volatile compounds in tea trees; however, there are significant differences between different elements in regulating the synthesis of volatile compounds in tea trees [24]. For example, Al and Pb are toxic and detrimental to the growth of tea trees, reducing their ability to synthesize volatile compounds [25,26], while Se and Mo are beneficial in increasing tea yield, volatile compound content, and tea aroma [27,28]. In this study, the volatile compounds of tea tree leaves were measured after treatment with different magnesium concentrations (Figure 1A) and it was found that a total of 772 volatile compounds was detected (Table S1), which could be categorized into 15 groups, namely, terpenoids (19.04%), esters (17.23%), ketones (12.44%), heterocyclic compounds (11.27%), alcohols (10.88%), aldehydes (5.83%), acids (5.18%), amines (3.63%), aromatics (3.37%), hydrocarbons (3.37%), phenols (2.85%), ethers (2.20%), nitrogen compounds (1.55%), halogenated hydrocarbons (0.65%), and sulfur compounds (0.52%), with the highest number of terpenoid compounds. Analysis of total volatile compounds after treatment with different magnesium concentrations revealed (Figure 1B) that ML, MM, and MH did not differ significantly (*p* > 0.05) in the total content of volatile compounds. The principal component analysis (Figure 1C) showed that principal component 1 and principal component 2 could effectively distinguish ML, MM, and MH in different regions, with an overall contribution of 68.35%. It can be seen that although there was no significant difference in the total content of volatile compounds in tea leaves after treatment with different magnesium concentrations, different groups of volatile compounds underwent significant changes in their content. Further analysis revealed (Figure 1D) that the volatile compounds most relevant to ML were mainly ketones, acids, halogenated hydrocarbons, alcohols, and aldehydes, while phenols were relevant to MM, and terpenoids, sulfur compounds, aromatics, and nitrogen compounds were relevant to MH. Qiu et al. [29] analyzed the effect of different amounts of applied nitrogen on the volatile compound content in tea leaves and found that an increase in nitrogen dosage had no significant effect on the total content of volatile compounds in tea leaves, but the terpenoids content was significantly increased. Xu et al. [10] analyzed the effect of magnesium fertilizer on the quality of tea leaves in the field and also found that an increase in the amount of magnesium had a lesser effect on the total content of volatile compounds in tea leaves. A similar phenomenon was also found in this study, which showed that magnesium regulation changed the content of different types of volatile compounds in tea tree leaves, although the effect on the total amount of volatile compounds in tea tree leaves was small.

### 3.2. Screening of Characteristic Volatile Compounds of Tea Tree Leaves Under Magnesium Regulation

**Figure 2:**
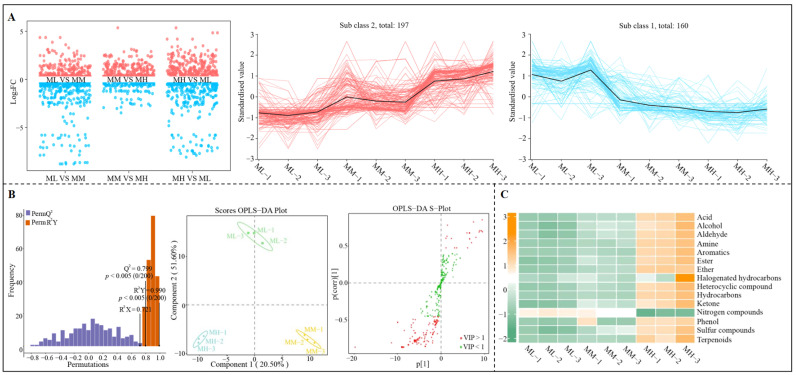
Screening and content analysis of key differential volatile compounds in tea tree leaves under magnesium regulation. Note: ML: Mg^2+^ concentration 0 mmol/L; MM: Mg^2+^ concentration 0.4 mmol/L; MH: Mg^2+^ concentration 0.8 mmol/L; (**A**) Volcano plot screening and K-Means clustering analysis of differential volatile compounds under magnesium regulation; (**B**) OPLS-DA model of ML, MM, and MH for screening of key differential volatile compounds; (**C**) Classification and content analysis of key differential volatile compounds.

**Figure 3:**
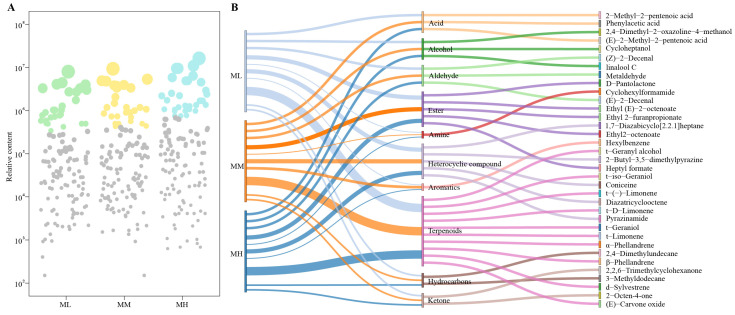
Screening and content analysis of characteristic volatile compounds of tea tree leaves under magnesium regulation. Note: ML: Mg^2+^ concentration 0 mmol/L; MM: Mg^2+^ concentration 0.4 mmol/L; MH: Mg^2+^ concentration 0.8 mmol/L; (**A**) Screening of characteristic volatile compounds under magnesium regulation by bubble feature map; (**B**) Classification of characteristic volatile compounds and their content analysis.

There are many types of volatile compounds in tea; however, the formation of its special odor characteristics depends on a small number of characteristic volatile compounds [30]. Shen et al. [31] found that the volatile compounds in An tea mainly consisted of esters, acids, and hydrocarbons, which accounted for more than 50% of the total volatile compounds, of which seven volatile compounds played a key role in the odor characteristics. Liang et al. [32] found that the key aroma active compounds in Wuyi rock tea were mainly 11 aromatic volatile compounds, especially linalool and geraniol. It can be seen that characteristic volatile compounds play an important role in the formation of special odor characteristics of tea. Accordingly, in this study, based on the determination of volatile compounds, firstly, multi-group volcano plots and K-means clustering analysis were used to screen for significant changes in differential volatile compounds in tea tree leaves at different magnesium concentrations. The results showed (Figure 2A) that a total of 357 volatile compounds significantly changed in tea tree leaves with increasing magnesium concentration, and subsequent K-means cluster analysis of these 357 volatile compounds revealed that 197 volatile compounds showed a significant upward trend and 160 showed a significant downward trend with increasing magnesium concentration. Based on the above significantly changed volatile compounds, OPLS-DA models of ML, MM, and MH were constructed to screen for key differential volatile compounds, and the results showed (Figure 2B) that the constructed OPLS-DA models effectively distinguished the three different treatments in different regions. The fit (R^2^Y = 0.990) and predictability (Q^2^ = 0.799) of the models reached a significant level (*p* < 0.005) and could be used to screen for key differential volatile compounds. From the S-Plot of the model, 130 key differential volatile compounds (VIP > 1) were derived that significantly differentiated ML, MM, and MH. Further analysis of the classification and content of key volatile compounds revealed (Figure 2C) that the 130 key volatile compounds could be classified into 15 groups, namely, acids, alcohols, aldehydes, amines, aromatics, esters, ethers, halogenated hydrocarbons, heterocyclic compounds, hydrocarbons, ketones, nitrogen compounds, phenols, sulfur compounds, and terpenoids, of which only the content of nitrogen compounds showed a decreasing trend as the magnesium concentration increased, while the content of the other 14 groups of volatile compounds showed an increasing trend. Key volatile compounds were analyzed using a bubble feature map, and 35 characteristic volatile compounds were screened and obtained (Figure 3A). The 35 characteristic volatile compounds could be mainly classified into 10 groups (Figure 3B), which were terpenoids (28.57%), ester (14.29%), heterocyclic compounds (14.29%), acids (8.57%), alcohols (8.57%), aldehydes (8.57%), hydrocarbons (5.71%), ketones (5.71%), amines (2.86%), and aromatics (2.86%), and in short, terpenoids accounted for the largest proportion, followed by esters and heterocyclic compounds.

The transformation relationship between compounds is extremely complex. For example, ketones, hydrocarbons, and aldehydes can be converted into alcohols, while alcohols and acids can form esters after esterification and may also be dehydrogenated to form isoprenoid structures, which in turn leads to the synthesis of terpenoids [33]. The conduct of these reactions involves the participation of a multitude of enzymes in a complex process [34]. As reported, terpenoids, esters, and heterocyclic compounds are the main components of volatile compounds in tea tree leaves, and they are also important volatile compounds for the formation of the special odor characteristics of tea, and their content directly affects the intensity of odor characteristics of tea [35,36]. It was evident that the key to the effect of magnesium regulation on volatile compounds in tea tree leaves was also focused on terpenoids, esters, and heterocyclic compounds, and changes in the content of these three groups of volatile compounds may affect the formation of odor characteristics of tea.

### 3.3. Contribution Weighting Analysis of Characteristic Volatile Compounds in Differentiating Between Different Magnesium Concentrations

**Figure 4:**
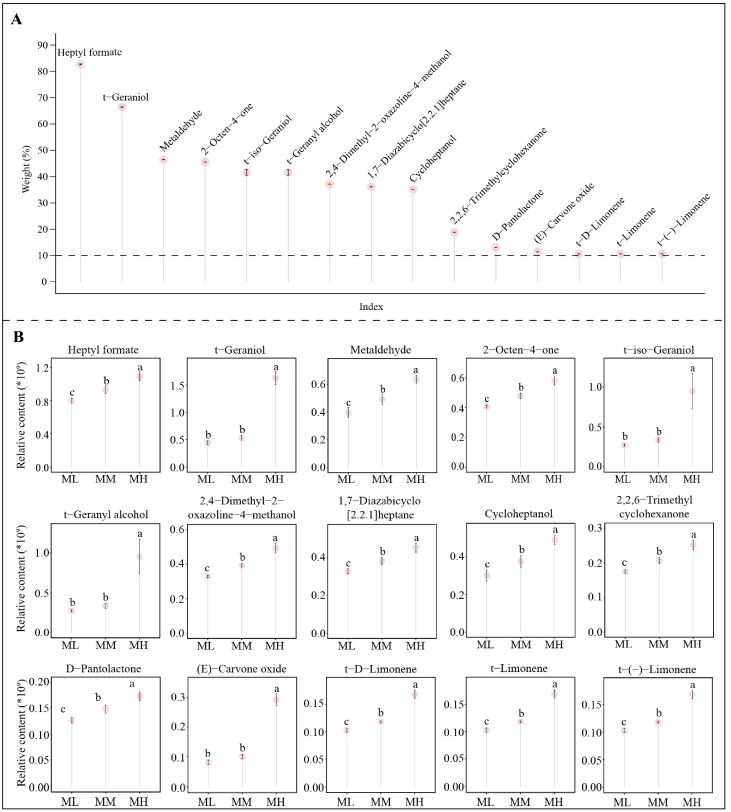
Contribution weights of characteristic volatile compounds and their content analysis under magnesium regulation. Note: ML: Mg^2+^ concentration 0 mmol/L; MM: Mg^2+^ concentration 0.4 mmol/L; MH: Mg^2+^ concentration 0.8 mmol/L; (**A**) TOPSIS analysis of the weights of characteristic volatile compounds in distinguishing between different magnesium concentrations; (**B**) Content analysis of characteristic volatile compounds with more than 10% of the contribution weight; different lower case letters indicate that the differences between different samples reached the *p* < 0.05 level.

On the basis of the previous analysis, this study further used TOPSIS weighting analysis to determine the contribution weights of the characteristic volatile compounds in distinguishing between different magnesium concentrations, and the results showed (Figure 4A) that for the 35 characteristic volatile compounds, only 15 of them had contribution weights greater than 10%, which were heptyl formate (83.20%), t-geraniol (66.50%), metaldehyde (46.78%), 2-octen-4-one (45.78%), t-geranyl alcohol (43.93%), t-iso-geraniol (43.93%), 2,4-dimethyl-2-oxazoline-4-methanol (37.31%), 1,7-diazabicyclo [2.2.1]heptane (36.30%), cycloheptanol (35.23%), 2,2,6-trimethylcyclohexanone (18.97%), D-pantolactone (13.13%), (E)-carvone oxide (11.34%), t-(-)-limonene (10.46%), t-D-limonene (10.46%), and t-limonene (10.46%). Moreover, the content of all the above 15 characteristic volatile compounds showed an increasing trend with the increase in magnesium concentration (Figure 4B). It was evident that after magnesium regulation, these 15 characteristic volatile compounds played a key role in distinguishing between different magnesium treatments, especially heptyl formate and t-geraniol, both of which were higher in contribution and content.

**Figure 5:**
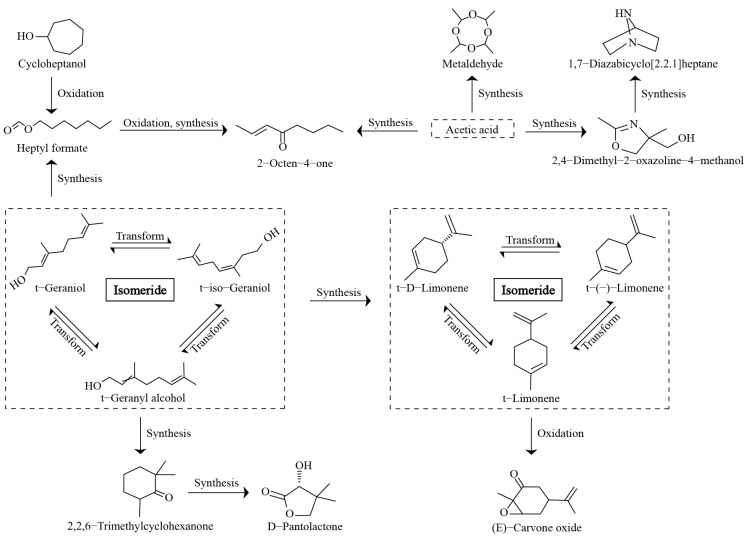
Transformation relationship of characteristic volatile compounds.

Further transformation analysis of the 15 characteristic compounds with high contribution rates revealed (Figure 5) that cycloheptanol could be converted to heptyl formate by oxidation, while heptyl formate could be converted to 2-octen-4-one by oxidation and synthesis. Secondly, acetic acid could synthesize three characteristic volatile compounds, such as 2-octen-4-one, metaldehyde, and 2,4-dimethyl-2-oxazoline-4-methanol, under suitable conditions, and 2,4-dimethyl-2-oxazoline-4-methanol was converted to 1,7-diazabicyclo [2.2.1]heptane by synthesis. Moreover, the transformation analysis of the characteristic compounds also revealed that t-geraniol, t-geranyl alcohol, and t-iso-geraniol were isomers of each other, which were converted to heptyl formate, 2,2,6-trimethylcyclohexanone, or isomers of limonene (t-limonene, t-(-)-limonene and t-D-limonene), whereas 2,2,6-trimethylcyclohexanone was converted by synthesis to D-pantolactone, and isomers of t-limonene were converted to (E)-carvone oxide by oxidation. It was evident that t-geraniol and its isomers played an important role in the formation of characteristic volatile compounds, which were precursors for the transformation of a large number of characteristic volatile compounds.

### 3.4. Odor Analysis of Characteristic Volatile Compounds of Tea Tree Leaves Under Magnesium Regulation

**Figure 6:**
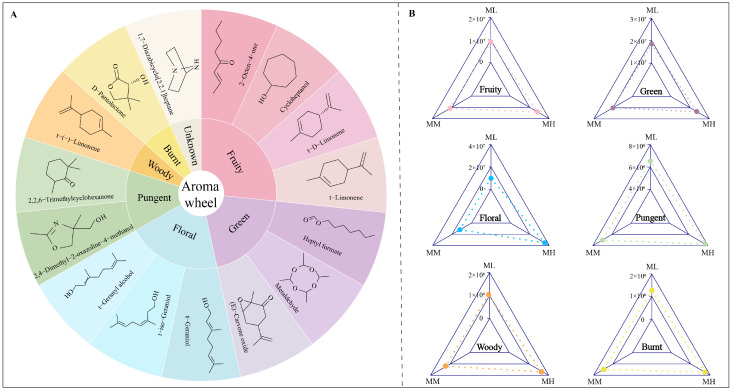
Effect of magnesium regulation on odor characteristics and their intensities in tea tree leaves. Note: ML: Mg^2+^ concentration 0 mmol/L; MM: Mg^2+^ concentration 0.4 mmol/L; MH: Mg^2+^ concentration 0.8 mmol/L; (**A**) Odor analysis of characteristic volatile compounds of tea tree leaves under magnesium regulation; (**B**) Intensity analysis of odor characteristics of tea under magnesium regulation.

The formation of odor characteristics of tea depends on the volatile compounds contained in tea, and different volatile compounds present different types of odor characteristics, and the intensity of odor characteristics is closely related to the content of volatile compounds [37,38]. For example, 2-octen-4-one has fruity odor characteristics [39], heptyl formate has green odor characteristics [40], geraniol has floral odor characteristics [41], 2,2,6-trimethylcyclohexanone has pungent odor characteristics [42], (-)-limonene has woody odor characteristics [43], and D-pantolactone has burnt odor characteristics [44]. Further analysis of the odor characteristics of the 15 characteristic volatiles in this study revealed (Figure 6A) that these 15 characteristic volatile compounds under magnesium regulation exhibited six main odor characteristics, namely fruity, green, floral, pungent, woody, and burnt. Among them, fruity was mainly contributed by 2-octen-4-one, cycloheptanol, t-D-limonene, and t-limonene; green was mainly contributed by heptyl formate, metaldehyde, and (E)-carvone oxide; floral was mainly contributed by t-geraniol, t-geranyl alcohol, and t-iso-geraniol; pungent was mainly contributed by 2,4-dimethyl-2-oxazoline-4-methanol and 2,2,6-trimethylcyclohexanone; and woody and burnt were mainly contributed by t-(-)-limonene and D-pantolactone(-), respectively. Further analysis of the intensity of the odor characteristics of tea leaves after magnesium regulation showed (Figure 6B) that, with the increase in magnesium concentration, the odor characteristics of fruity, green, floral, pungent, woody, and burnt in tea were all significantly enhanced, especially floral, fruity, and green. It was evident that magnesium was beneficial to improving the intensity of odor characteristics of tea.

## 4. Conclusions

**Figure 7:**
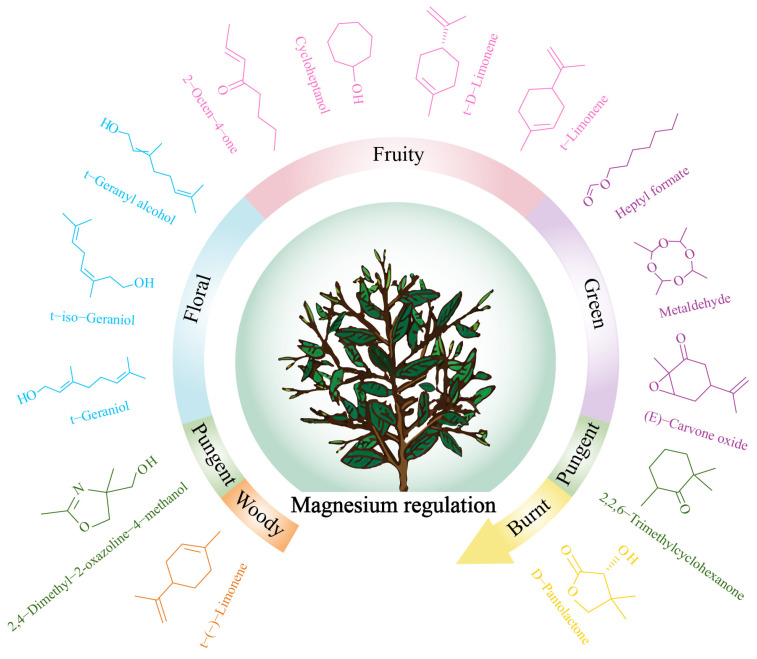
Effect of magnesium regulation on characteristic volatile compounds and odor characteristics of tea tree leaves.

In this study, the effect of magnesium regulation on volatile compounds and odor characteristics of tea tree leaves was analyzed, and the results showed (Figure 7) that magnesium regulation significantly changed the content of volatile compounds in tea tree leaves, especially terpenoids, esters, and heterocyclic compounds. After magnesium regulation, the characteristic volatile compounds that changed significantly in tea tree leaves were mainly 15 kinds, and their contents showed an increasing trend with the increase of magnesium ion concentration. Among them, heptyl formate and t-geraniol played a key role in distinguishing between different concentrations of magnesium ions. After magnesium regulation, the characteristic volatile compounds of tea tree leaves mainly exhibited five types of odor characteristics, such as fruity, green, floral, pungent, woody, and burnt, the intensities of which all showed a significant upward trend with the increase of magnesium concentration, especially floral, fruity, and green. In conclusion, magnesium is beneficial to enhancing the synthesis of volatile compounds in tea leaves and the intensity of odor characteristics such as floral, fruity, and green. This study provides an important theoretical basis for the application of exogenous magnesium ions to regulate the growth of tea trees and improve the aroma quality of tea leaves.

## Data Availability

The original contributions presented in the study are included in the article/Supplementary Material, further inquiries can be directed to the corresponding authors.

## Supplementary Materials

The following supporting information can be downloaded at: https://www.mdpi.com/article/10.3390/foods14061043/s1, Table S1. Raw data of volatile compounds in tea leaves.
